# Population-Specific Pharmacogenomic Profiling of *NAT2*, *CYP2E1*, and *SLCO1B1* in Tuberculosis Patients from Southern Peru: A Feasibility Pilot Study

**DOI:** 10.3390/jpm16040184

**Published:** 2026-03-29

**Authors:** Tatiana Chavez-Arias, Cecilia Manrique-Sam, Yuma Ita-Balta, Edgar Montánchez-Carazas, Alexis Germán Murillo Carrasco, Miguel Farfán-Delgado

**Affiliations:** 1 Escuela de Postgrado, Universidad Católica de Santa María, Arequipa 04013, Peru; tchavez@ucsm.edu.pe; 2 Área de Histocompatibilidad y Biología Molecular, Hospital Nacional Carlos Alberto Seguin Escobedo, Arequipa 04001, Peru; 3 Escuela de Medicina Humana, Universidad Católica de Santa María, Arequipa 04013, Peru; mmanriques@ucsm.edu.pe (C.M.-S.); emontanchez@ucsm.edu.pe (E.M.-C.); mfarfan@ucsm.edu.pe (M.F.-D.); 4 Pathogen Genetics Research Group (PATHO-GEN), Organization for Medical Innovation and Collaboration for Science (OMICS), Lima 15001, Peru

**Keywords:** tuberculosis, pharmacogenomics, polymorphisms, *NAT2*, *CYP2E1*, *SLCO1B1*, isoniazid, rifampicin

## Abstract

Tuberculosis (TB) remains a major public health challenge in Peru, where interindividual variability in treatment response and drug-induced hepatotoxicity may be influenced by host genetic background. This study aimed to characterize clinically relevant polymorphisms in *NAT2*, *CYP2E1*, and *SLCO1B1* in a cohort of TB patients from Southern Peru, a genetically underrepresented Andean population. Thirty-five adults receiving first-line therapy (isoniazid and rifampicin) underwent targeted Sanger sequencing of key functional variants among these three genes. *NAT2* acetylator phenotypes were predominantly intermediate (68.6%), followed by rapid (20%) and slow (11.4%) profiles, with high minor allele frequencies for rs1041983 and rs1801280. *CYP2E1* functional promoter variants were infrequent, whereas *SLCO1B1* exhibited notable allelic heterogeneity, suggesting potential variability in rifampicin transport. Comparative analysis with previously reported Peruvian data revealed regional differences in acetylator distribution, supporting population-specific pharmacogenomic stratification. Although clinical toxicity outcomes were not evaluated, the high prevalence of reduced acetylation genotypes suggests a substantial proportion of patients may benefit from genotype-informed isoniazid dosing strategies. These findings provide foundational data for implementing precision medicine approaches using affordable and targeted technologies in TB management within Andean populations and support the integration of pharmacogenomics into national TB control programs.

## 1. Introduction

Tuberculosis (TB) remains a leading cause of infectious disease-related mortality globally. In 2022, the World Health Organization (WHO) reported over 10 million new cases and 1.3 million deaths, surpassing the mortality burden of HIV/AIDS and malaria [1]. In Peru, TB is a priority public health issue, with approximately 27,000 new cases annually, including more than 1500 cases of multidrug-resistant (MDR) TB and nearly 100 cases of extensively drug-resistant (XDR) TB, which complicates control and treatment strategies [2].

The standard treatment for drug-susceptible TB combines isoniazid (INH), rifampicin (RIF), ethambutol, and pyrazinamide for at least six months. While effective, this regimen is associated with significant adverse effects, particularly anti-tuberculosis drug-induced hepatotoxicity (ATDH), which can affect between 2% and 28% of patients [3,4]. ATDH not only compromises treatment safety but also promotes non-adherence, increasing the risk of therapeutic failure, relapse, and resistance.

Host genetic variability is a determining factor in the response to anti-TB drugs. Polymorphisms in genes such as *NAT2* and *CYP2E1* modify the biotransformation of INH and are associated with slow acetylation phenotypes, which increase the risk of hepatotoxicity [5]. Likewise, variants in transporter genes such as *SLCO1B1* can alter the pharmacokinetics of RIF, influencing therapeutic efficacy and susceptibility to toxicity [6]. Studies in Asian and Latin American populations have shown high frequencies of intermediate and slow *NAT2* acetylators, while rapid acetylators predominate in European populations [7,8]. However, data in Peru are limited and mostly restricted to cohorts from Lima, without representative information from other regions [8,9].

Although pharmacogenetic studies of *NAT2*, *CYP2E1*, and *SLCO1B1* have been previously conducted in Peruvian tuberculosis patients from Lima, the genetic diversity of the Peruvian population is known to vary significantly across geographical regions. Southern Peru, particularly the Andean region, presents a distinct genetic and demographic structure shaped by historical north–south population dynamics, admixture patterns, and a higher proportion of Andean ancestry compared to the capital city. Recent population genomics studies have demonstrated that Peruvian urban populations exhibit marked regional genetic differentiation, with southern populations remaining underrepresented in genomic and pharmacogenetic research. Given that polymorphisms in genes involved in anti-tuberculosis drug metabolism and transport may differ in frequency across subpopulations, region-specific data are essential to support the implementation of personalized treatment strategies.

In this context, the present study aimed to characterize *NAT2*, *CYP2E1*, and *SLCO1B1* variants in tuberculosis patients from Southern Peru, providing baseline pharmacogenomic data for a population that has not been previously investigated.

## 2. Materials and Methods

### 2.1. Study Design and Participants

An observational, descriptive pilot study was conducted in adult patients with confirmed pulmonary tuberculosis from Southern Peru (Arequipa). A total of 40 patients were initially recruited with the following inclusion criteria: age ≥ 18 years, confirmed pulmonary tuberculosis by positive sputum smear or culture for Mycobacterium tuberculosis, and receipt of first-line anti-tuberculosis therapy including INH and RIF. Patients with prior anti-tuberculosis treatment or uncontrolled HIV co-infection were excluded.

Five samples were excluded from molecular analysis due to insufficient DNA quality, resulting in a final cohort of 35 patients. The analyzed cohort consisted of 60% males and 40% females, with a mean age of 32 years.

### 2.2. Sample Size

This study was conceived as a pilot, observational, and descriptive pharmacogenetic investigation aimed at establishing baseline allele and phenotype frequencies in a previously underrepresented population from Southern Peru. The sample size was determined by feasibility within the defined recruitment period (September 2024 to February 2025), with the primary objective of generating preliminary allele and phenotype frequency estimates rather than conducting hypothesis-driven inferential analyses. Accordingly, the study was not powered to detect genotype–clinical outcome associations.

### 2.3. Sample Collection and DNA Extraction

Three milliliters of peripheral blood were collected in ethylenediaminetetraacetic acid (EDTA). Genomic DNA was extracted from 200 µL of the buffy coat using the NZY Tissue gDNA Isolation kit (NZYTech, Lisbon, Portugal) according to the manufacturer’s instructions. DNA quality and concentration were evaluated with an Implen NanoPhotometer™ NP80 spectrophotometer (Implen, Munich, Germany) at 260 and 280 nm. Only samples with a 260/280 ratio between 1.6 and 1.9 were processed further.

### 2.4. Amplification and Sequencing

The gene regions of interest (*NAT2*, *CYP2E1*, and *SLCO1B1*) were amplified using endpoint PCR with Platinum Taq DNA Polymerase (Invitrogen, Carlsbad, CA, USA). Specific primers, previously designed and verified with NCBI reference sequences (*NAT2*: NG_012246.1; *CYP2E1*: NG_008383.1; *SLCO1B1*: NG_011745.1), were used according to their chromosomal location:

(*CYP2E1* Forward: 5′-AACGCCCCTTCTTGGTTCAG-3′;
*CYP2E1* Reverse: 5′-ACAGACCCTCTTCCACCTTCTA-3′;
*NAT2* Forward: 5′-TAACATGCATTGTGGGCAAGC-3′;
*NAT2* Reverse: 5′-TGGGTGATACATACACAAGGG-3′;
*SLCO1B1* Forward: 5′-GGGGAAGATAATGGTGCAAA-3′;
*SLCO1B1* Reverse: 5′-CATCCAGTTCAGATGGACAAAA-3′).

Reactions were performed in a S1000 Thermal Cycler (BioRad, Hercules, CA, USA) under the following cycling conditions: initial denaturation at 95 °C for 5 min, followed by 35 cycles of denaturation at 95 °C for 30 s, annealing at 58 °C for 30 s, and extension at 72 °C for 45 s, with a final extension at 72 °C for 7 min. Amplicons were analyzed by 2% agarose gel electrophoresis with a 100 bp molecular weight marker to verify specificity and expected size. Products were sequenced using the Sanger method (BigDye Terminator v3.1, ThermoFisher Scientific, Foster City, CA, USA). Sequences were analyzed on both strands and compared with GenBank references.

### 2.5. Sanger Sequencing Data Processing and Base-Calling

Initial processing of .ab1 electropherogram files was performed using the sangerseqR package (R v.4.5). This package reads the .ab1 file and performs base-calling using the makeBaseCalls function. To detect potential variants, a secondary peak recognition threshold (secondary_ratio) was set to 0.5. This means any secondary peak with 50% or more of the height of the primary peak was considered a potential alternate allele. Nucleotide combinations were encoded using IUPAC ambiguity codes (e.g., A and C were encoded as M).

### 2.6. Consensus Sequence Generation and Alignment

Following base-calling, a consensus sequence was generated for each sample using the consensus_two_calls function. This algorithm integrates both primary and secondary bases. The primary base is prioritized unless the secondary base has a significantly higher confidence score (a difference of more than 0.2 on a 0–1 scale). If different bases have similar confidence scores, IUPAC ambiguity codes are applied. Bases with confidence scores below a pre-established threshold (0.5) were designated as N (unidentified base).

All alignment files were manually reviewed and curated. The final alignment was saved as a FASTA-formatted .aln file for downstream analysis.

Although *NAT2* is a highly polymorphic gene, the selection of five key single-nucleotide polymorphisms (SNPs) in this study was based on their established relevance for acetylator phenotype assignment and their high frequency in diverse populations. These variants capture the most common *NAT2* haplotypes (*4, *5, *6, and *7) that together account for the vast majority of acetylator phenotypes reported worldwide.

Given the pilot and descriptive nature of this study, the use of a reduced SNP panel was considered appropriate to generate baseline pharmacogenetic data in an underrepresented population. While extended SNP panels may provide finer resolution, the selected variants are sufficient for reliable acetylator phenotype classification in population-based studies and have been widely used in similar pharmacogenetic investigations.

### 2.7. Variant Annotation and Visualization

The identified variants and their associated genotypes were compiled into a VCF (Variant Call Format) file (v4.2). The file included chromosomal position, reference and alternative alleles, AF, and genotype for each sample. The VCF file was submitted to the Ensembl Variant Effect Predictor (VEP). This tool provided allele frequency information from databases like gnomAD and the 1000 Genomes Project, as well as pathogenicity predictions and dbSNP IDs (rs identifiers). Data from the Ensembl VEP output were parsed to extract relevant information, including allele frequency (AF=) from our cohort and rs identifiers. For focused analysis, only variants with an AF of ≥0.1 were included. For comparative visualization, allele frequencies from external databases were extracted from the INFO column, specifically gnomADg_AMR_AF (for the American population in gnomAD) and AMR_AF (for the American population in the 1000 Genomes Project).

### 2.8. Statistical Analysis and Hardy–Weinberg Equilibrium

Statistical analyses were limited to descriptive summaries of genotype, allele, haplotype, and phenotype frequencies. Hardy–Weinberg equilibrium (HWE) was evaluated for each variant as part of genotype-level quality control. Genotype data were extracted from the VCF-derived matrix, excluding non-genotype fields (chromosome, position, variant identifiers, reference and alternate alleles, quality metrics, and metadata). For each variant, individual genotypes coded as missing (./.) were excluded from analysis. Variants with fewer than five non-missing genotype calls were not tested for HWE due to insufficient sample size. Genotype counts were obtained as the number of homozygous reference (0/0; nAA), heterozygous (0/1; nAB), and homozygous alternate (1/1; nBB) individuals. Deviation from the Hardy–Weinberg equilibrium was assessed using the exact test of HWE, as implemented in the HWExact function from the HardyWeinberg R package. This method is appropriate for small sample sizes and unbalanced genotype distributions. The resulting exact test *p*-value was recorded for each variant.

Variants lacking valid HWE *p*-values were excluded from downstream analyses. In addition, variants showing extreme deviation from HWE (*p* < 1 × 10^−6^) were flagged and removed as likely technical artifacts. To evaluate the global distribution of HWE *p*-values and assess systematic inflation or deflation, a quantile–quantile (Q–Q) plot was generated by comparing observed versus expected −log_10_(*p*) values under the null hypothesis of Hardy–Weinberg equilibrium.

### 2.9. Variant Filtering, Genotype-Based Clustering, and Haplotype Validation

Variants included in the genotype-based clustering analysis were selected through a structured pipeline aimed at retaining informative loci while minimizing technical artifacts. Hardy–Weinberg equilibrium (HWE) was assessed for all variants, and loci showing extreme deviation from equilibrium (*p* < 1 × 10^−10^) were excluded.

Genotype calls were extracted and standardized to canonical VCF formats (0/0, 0/1, 1/1, ./.). Variant-level call rate and heterozygosity rate were computed across samples to identify and exclude loci with aberrant genotype distributions. To ensure sufficient population-level informativeness, only variants with a minor allele frequency (MAF) greater than 10% were retained. Variants were annotated by gene based on chromosomal location (*CYP2E1*, *NAT2*, and *SLCO1B1*), and rsIDs were assigned when available.

For each gene, genotype matrices were constructed, and genotypes were numerically encoded (0 = homozygous reference, 1 = heterozygous, 2 = homozygous alternate; missing = NA). Samples and variants with insufficient genotype completeness were removed before clustering. Hierarchical clustering of both variants and samples was then performed to identify groups of individuals sharing similar multi-locus genotype profiles, representing haplotype-like patterns. These patterns were visualized using heatmaps generated with the pheatmap R package.

To validate the inferred haplotype-like structures, filtered variants were exported to VCF format and subjected to haplotype phasing using Beagle (version 27Feb25.75f), without genotype imputation. Phased genotypes were decomposed into individual haplotypes and summarized at the gene level. The resulting phased haplotypes were compared with genotype-based clusters, confirming concordance between visually inferred genotype groupings and statistically phased haplotypic configurations.

### 2.10. Ethical Considerations

The study was approved by the Institutional Ethics Committee for Research of the Arequipa Healthcare Network—EsSalud (Arequipa, Peru) under NIT N° 1161-2024-165, issued on 25 July 2024.

## 3. Results

### 3.1. Study Participants

A total of 40 adult patients with confirmed tuberculosis were recruited for the study. Five patients were excluded from the molecular analysis due to insufficient DNA quality, resulting in a final analyzed sample of 35 patients. The cohort was composed of 60% males and 40% females, with a mean age of 32 years. All participants were receiving first-line anti-tuberculosis treatment, including isoniazid (INH) and rifampicin (RIF), at the time of sample collection and had no documented history of chronic liver disease or previous anti-tuberculosis treatment. Routine liver enzyme measurements were not systematically collected as part of the study design and were therefore not available for analysis.

### 3.2. Hardy–Weinberg Equilibrium

**Figure 1:**
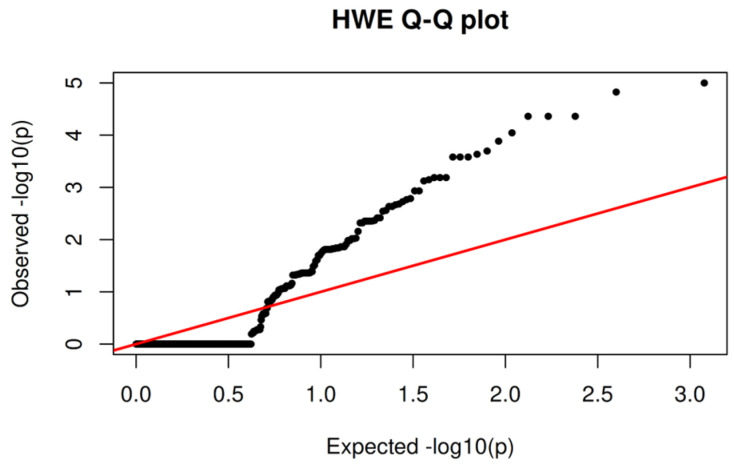
Hardy–Weinberg equilibrium Q–Q plot in a clinically selected cohort. Quantile–quantile (Q–Q) plot of observed versus expected −log_10_(*p*) values from exact Hardy–Weinberg equilibrium tests across all analyzed variants. The red diagonal line represents the null expectation under the Hardy–Weinberg equilibrium.

The distribution of Hardy–Weinberg equilibrium (HWE) *p*-values was examined using a quantile–quantile (Q–Q) plot comparing observed versus expected −log_10_(*p*) values under the null hypothesis of Hardy–Weinberg equilibrium (Figure 1).

Overall, the majority of variants followed the expected null distribution at low to moderate significance levels, indicating no systematic inflation of HWE test statistics across the dataset. However, a progressive upward deviation from the diagonal was observed in the upper tail of the distribution, reflecting an excess of variants with small HWE *p*-values.

Given that the analyzed cohort consists of clinically selected patients rather than a random population sample, deviations from the Hardy–Weinberg equilibrium are not unexpected. Such departures may arise from population structure, non-random sampling, or selection related to disease status or pharmacogenetically relevant traits, in addition to potential technical or genotyping-related artifacts. Variants exhibiting extreme deviation from equilibrium (*p* < 1 × 10^−6^) were therefore flagged and excluded from downstream analyses as part of a conservative genotype quality control strategy, while moderate deviations were retained and interpreted in the context of the study design.

### 3.3. NAT2 Pharmacogenetic Findings

**Figure 2:**
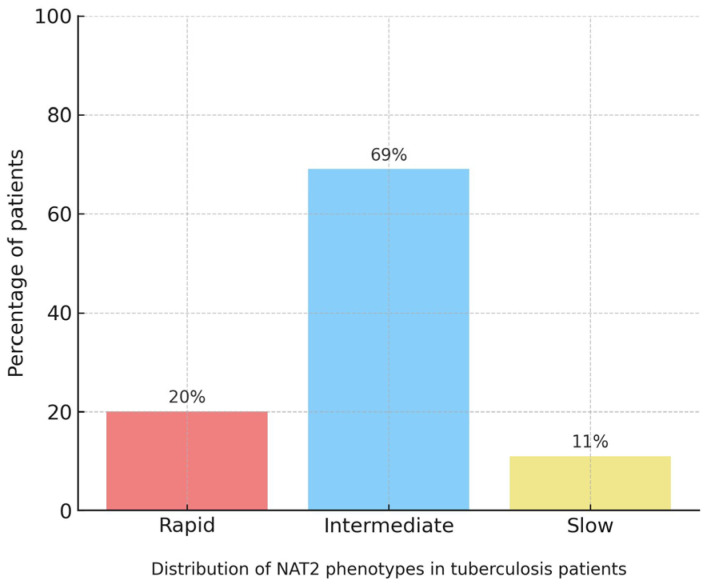
Distribution of *NAT2* Acetylator Phenotypes in 35 TB Patients from Southern Peru. The blue bar represents the proportion of intermediate acetylator individuals (approx. 69%), the green bar represents rapid acetylators (20%), and the orange bar represents slow acetylators (11%). A marked predominance of the intermediate phenotype is observed, followed by a minority of rapid and slow phenotypes.

Five main *NAT2* variants were identified: rs1041983, rs1801280, rs1799929, rs1208, and rs1799931. The combination of these SNPs allowed for the determination of acetylator phenotypes: 68.6% intermediate, 20% rapid, and 11.4% slow (Figure 2). The allelic frequency of SNP rs1041983 (C282T) was 34%, while rs1801280 (T341C) reached 18%. These findings confirm a high proportion of alleles associated with reduced acetylation in the studied population.

**Figure 3:**
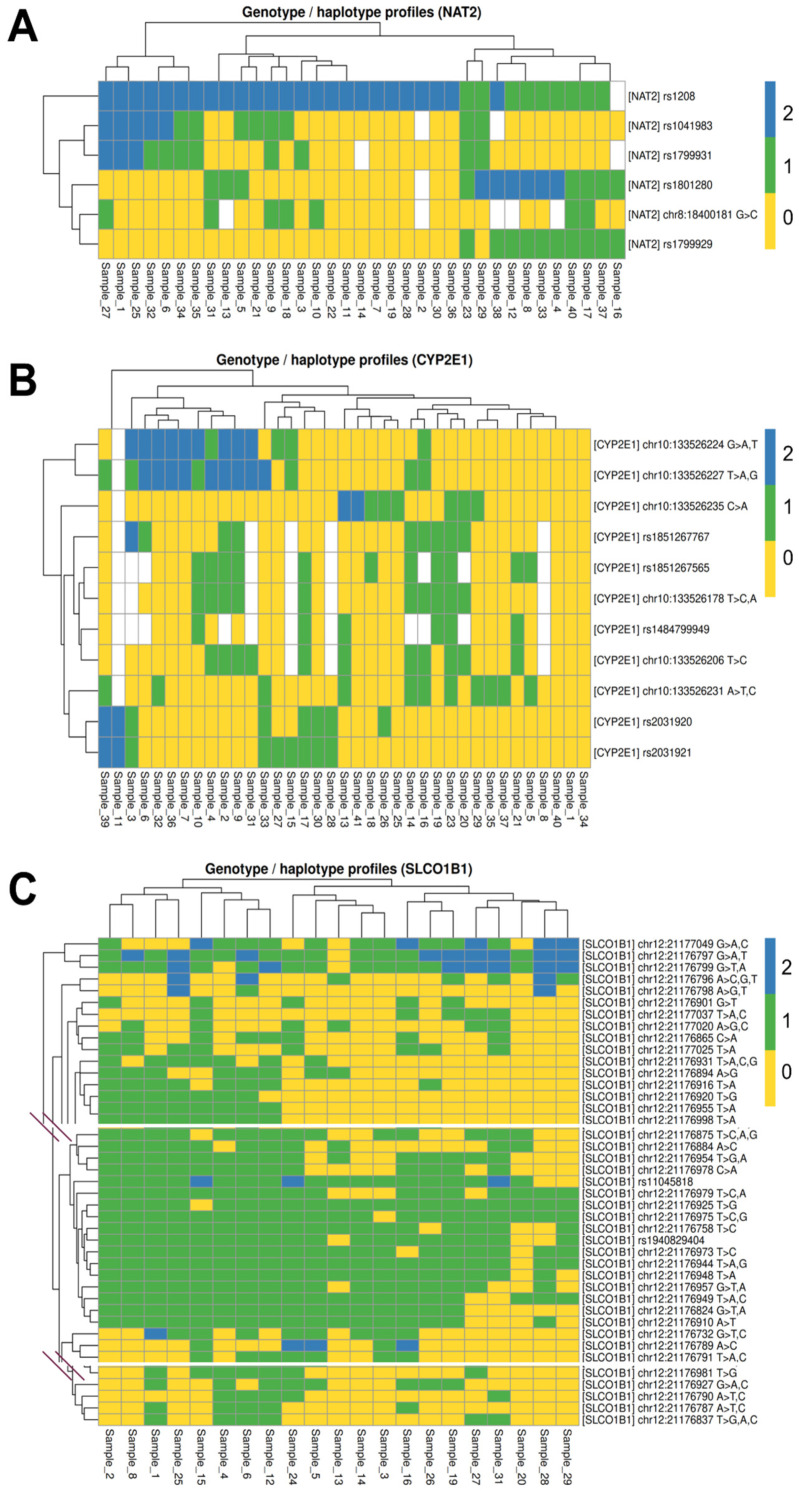
Genotype and inferred haplotype profiles of pharmacogenes. Heatmaps show individual-level genotype profiles for *NAT2* (**A**), *CYP2E1* (**B**), and *SLCO1B1* (**C**) variants. Rows represent single-nucleotide polymorphisms (SNPs), ordered by hierarchical clustering based on genotype similarity, while columns correspond to individual samples. Genotypes are color-coded according to allele dosage: 0 = homozygous reference (yellow), 1 = heterozygous (green), and 2 = homozygous variant (blue); missing genotypes are shown in white. Dendrograms illustrate hierarchical clustering of both samples and variants, revealing recurrent genotype-based clusters consistent with shared haplotypic structures. The panel for SLCO1B1 variants was cropped due to the high number of variants found. The uncropped figure can be found in Figure S1.

Hierarchical clustering of genotype profiles (Figure 3A) revealed the presence of distinct genotype-based clusters, supported by these common variants plus an unannotated variant (chr8:18400181G>C), suggesting non-random co-segregation of *NAT2* variants and the existence of recurrent haplotypic structures. One major cluster was characterized by the predominance of wild-type or low-impact alleles, consistent with rapid acetylator-associated STAR alleles such as NAT2*4 or NAT2*12. In contrast, other clusters showed enrichment of variant combinations involving rs1041983, rs1801280, and rs1799931, which are compatible with reduced-function STAR alleles, including NAT2*5, NAT2*6, and NAT2*7, either in homozygous or compound heterozygous configurations.

From a pharmacogenetic perspective, these genotype clusters align with ClinPGx-defined acetylator categories, reinforcing the biological plausibility of the inferred phenotypes. Individuals clustering with multiple reduced-function alleles are likely to exhibit slower drug metabolism, which has clinical implications for drugs metabolized by *NAT2*, such as isoniazid, sulfonamides, and certain aromatic amines. Conversely, clusters enriched in reference or high-activity alleles are consistent with rapid acetylation profiles and potentially lower drug exposure.

### 3.4. CYP2E1 Pharmacogenetic Findings

Regarding *CYP2E1* polymorphisms, five main variants were detected, including rs2031920 and rs2031921 in the promoter region. The majority of patients (62–86% depending on the SNP) had a wild-type genotype, with heterozygotes at 11–38% and homozygous mutants at <5%. This indicates that functional variants of *CYP2E1* are infrequent in this cohort.

Hierarchical clustering of genotype profiles (Figure 3B) revealed the presence of distinct genotype-based clusters, reflecting non-random co-segregation of *CYP2E1* variants. One major cluster was characterized by the co-occurrence of promoter-region variants, particularly rs2031920 and rs2031921, which are known to influence *CYP2E1* transcriptional activity. In contrast, other clusters showed largely reference genotypes across all analyzed loci, consistent with canonical *CYP2E1* haplotypes.

From a pharmacogenetic standpoint, these genotype clusters are compatible with established *CYP2E1* STAR allele groupings, such as CYP2E1*1 [10] and variant haplotypes related to CYP2E1*5. The observed clustering suggests potential differences in *CYP2E1* expression and metabolic capacity among individuals, which may have implications for the biotransformation of xenobiotics and drugs metabolized by this enzyme.

### 3.5. SLCO1B1 Pharmacogenetic Findings

In *SLCO1B1*, a heterogeneous genetic profile was observed, with multiple SNPs distributed along the analyzed fragment. Genotype frequencies ranged from 60–80% for wild-type homozygotes, 15–40% for heterozygotes, and 2–15% for homozygous mutants, depending on the SNP. Some synonymous variants reached high allelic frequencies (~80%), suggesting the existence of specific haplotypes in this population.

Hierarchical clustering of *SLCO1B1* genotype profiles (Figure 3C) revealed distinct genotype-based clusters, indicating structured co-inheritance of *SLCO1B1* variants and recurrent haplotypic patterns. One major cluster was characterized by near-complete reference genotypes, consistent with normal-function haplotypes typically associated with SLCO1B1*1 alleles. In contrast, other clusters showed enrichment of multiple variant loci, including combinations compatible with reduced- or decreased-function STAR alleles, such as SLCO1B1*5-, SLCO1B1*15-, or SLCO1B1*17-related haplotypes.

From a clinical pharmacogenetics perspective, these genotype clusters align with ClinPGx-defined transporter phenotypes, distinguishing individuals with normal versus reduced OATP1B1 transport activity. Such differences are clinically relevant for drugs that rely on OATP1B1-mediated hepatic uptake, including statins and other commonly prescribed agents, and may contribute to inter-individual variability in drug exposure, and toxicity risk. Collectively, these findings support the relevance of *SLCO1B1* haplotypic stratification for pharmacogenetic interpretation in the studied population.

### 3.6. Overall Observations

These results indicate that *NAT2* acetylation phenotypes are distributed similarly among the different types of clinical tuberculosis, with no association pattern observed. This is consistent with the biological function of *NAT2*, whose genetic variability is directly related to the rate of isoniazid metabolism and, therefore, to the efficacy and safety of pharmacological treatment, but does not intervene in the pathogenic mechanisms that determine the anatomical location of the infection. Consequently, *NAT2* genotyping has clinical value in therapeutic personalization and the prevention of hepatotoxicity, but it does not serve as a predictor of the clinical form of tuberculosis a patient will develop.

To contextualize the pharmacogenetic profiles observed in this study, a descriptive comparison with previously published data from a tuberculosis cohort in Lima is presented in Table 1.

In the southern Peru cohort (n = 35), *NAT2* acetylator phenotypes were predominantly intermediate (24/35, 68.6%), followed by rapid (7/35, 20%) and slow acetylator phenotypes (4/35, 11.4%). A descriptive comparison with a previously reported cohort from Lima is presented in Table 1.

Analysis of the minor allele frequency (MAF) of *NAT2* gene polymorphisms revealed a high prevalence of genetic variants associated with reduced acetylation capacity in the analyzed cohort. In particular, the rs1041983 (C282T) and rs1801280 (T341C) polymorphisms, both involved in the formation of slow-acetylating *NAT2* haplotypes, showed MAFs of 0.27 and 0.29, respectively (Table 2).

Meanwhile, the rs1208 (A803G) polymorphism showed a high alternative allele frequency (AF = 0.87); however, its MAF was low (0.13), reflecting that this allele is the major allele in this population. In contrast, the rs1799929 and rs1799931 polymorphisms presented intermediate MAFs (0.14 and 0.21, respectively), further contributing to the genetic heterogeneity of the *NAT2* gene observed in the studied cohort.

## 4. Discussion

This study characterized polymorphisms in *NAT2*, *CYP2E1*, and *SLCO1B1* in patients with pulmonary tuberculosis from Southern Peru (Arequipa). The findings show a predominance of intermediate and slow acetylator phenotypes determined by *NAT2* variants, a low prevalence of functional polymorphisms in *CYP2E1*, and marked genetic heterogeneity in *SLCO1B1* within the studied population.

Regarding the NAT2 gene, acetylator phenotypes in Southern Peru were predominantly intermediate (24/35, 68.6%), followed by rapid (7/35, 20.0%) and slow (4/35, 11.4%). This distribution differs substantially from that reported in a Lima cohort where slow acetylators represented 47.5% and intermediate phenotypes 37.7%. These findings indicate marked intra-country pharmacogenetic heterogeneity [7].

The observed minor allele frequencies of rs1041983 (MAF = 0.27), rs1801280 (MAF = 0.29), and rs1799931 (MAF = 0.21) are consistent with reduced-function haplotypes corresponding to NAT2*5-, NAT2*6-, and NAT2*7-related star alleles. However, the relatively lower prevalence of homozygous reduced-function combinations likely explains the moderate proportion of slow acetylators (11.4%) observed in this cohort.

Clinically, slow acetylators are known to have an increased risk of isoniazid-induced hepatotoxicity due to accumulation of toxic metabolites, as supported by meta-analytic evidence [12,13]. In our cohort, no association was observed between *NAT2* phenotype and clinical location of TB, which is biologically plausible given that *NAT2* influences INH pharmacokinetics rather than pathogenic mechanisms of infection.

Although the observed difference in slow acetylator frequency between Southern Peru (11.4%) and Lima (47.5%) appears notable, this study was not designed to formally assess population stratification or genetic differentiation. Several factors may contribute to this discrepancy, including differences in ancestral composition between regions, sampling variability due to the limited cohort size, and potential demographic differences in recruitment settings. Southern Peru has a distinct historical and migratory background compared with the capital region, which may influence allele distribution patterns.

Most patients had the wild-type *CYP2E1* (promoter) genotype, a finding consistent with reports in non-Asian populations [8]. Although its isolated clinical impact is limited, this gene encodes an enzyme that participates in the generation of reactive metabolites and reactive oxygen species during INH biotransformation. Consequently, a combination of *CYP2E1* variants (NAT2*5 and NAT2*6) with a slow *NAT2* phenotype has been associated with a higher risk of hepatotoxicity [14].

Our results support this hypothesis and suggest that *CYP2E1* could act as a risk modulator in specific subgroups. The analysis of *SLCO1B1* revealed a wide genetic diversity, with intermediate to high frequencies for several variants. This is relevant because functional polymorphisms in this gene, such as *SLCO1B1* c.521T>C (rs4149056), have been associated with increased exposure to rifampicin [6]. While this specific SNP was not detected in all patients, other identified variants could influence RIF pharmacokinetics, as observed in Asian populations [15]. These findings suggest that genetic variability in *SLCO1B1* might, at least in part, explain inter-individual differences in rifampicin tolerance and efficacy in the Peruvian population.

Although this study provides baseline pharmacogenetic profiling of *NAT2*, *CYP2E1*, and *SLCO1B1* in a previously underrepresented Andean population, some limitations must be acknowledged. First, the relatively small sample size (n = 35) limits the precision of frequency estimates and precludes robust inferential analyses or stratified subgroup comparisons. As previously discussed in the context of genetic polymorphism studies [16,17], adequately powered cohorts are essential for detecting modest genotype–phenotype effects and for conducting reliable inter-population comparisons. Nevertheless, given the exploratory nature of the present study, the cohort size was not intended to support formal association testing or definitive conclusions regarding regional genetic differentiation. Rather, the primary objective was to establish preliminary allele and phenotype frequency data in a previously underrepresented population. Confirmation of these patterns and assessment of their clinical implications will require larger, multicenter studies incorporating pharmacokinetic and outcome-based endpoints.

Second, no genotype–phenotype associations or clinical toxicity endpoints were evaluated in this pilot study. Hepatotoxicity biomarkers, such as liver enzyme levels, and detailed clinical follow-up data were not systematically collected as the study was specifically designed as a pharmacogenetic frequency characterization. Third, pharmacokinetic parameters, including plasma drug concentrations, clearance rates, or exposure metrics, were not measured, preventing direct assessment of the functional impact of the identified variants.

The analysis was also restricted to selected regions within three genes, excluding other loci potentially relevant to anti-tuberculosis drug metabolism and transport. Consequently, the present findings should be interpreted as preliminary population-level pharmacogenetic data intended to inform the design of larger, adequately powered studies incorporating pharmacokinetic measurements and clinical outcomes.

From a biological standpoint, *NAT2* acetylator status was not associated with tuberculosis clinical presentation, which is consistent with its established role in drug metabolism rather than disease pathogenesis. Although clinical outcomes were not evaluated, the observed frequency of slow acetylator genotypes (11.4%) suggests that a proportion of patients may be at increased risk of altered isoniazid exposure, warranting further investigation in prospective outcome-based studies.

Moreover, our study represents one of the first pharmacogenetic approaches in TB patients from Southern Peru using affordable and targeted technologies. Our results confirm the high prevalence of *NAT2* intermediate/slow acetylators in this population, as well as the genotypic diversity in *SLCO1B1*. This supports the need to consider pharmacogenomics in designing personalized anti-TB therapeutic strategies [18]. In resource-limited settings, *NAT2* genotyping could be a cost-effective tool for identifying patients at higher risk of INH-induced hepatotoxicity, while characterizing *SLCO1B1* variants could guide future studies on optimal individual RIF dosing.

The distribution of allele frequencies observed in the *NAT2* gene is consistent with previous studies in Latin American and Andean populations, which have reported a predominance of alleles associated with intermediate and slow acetylation. The high MAF of rs1041983 and rs1801280 supports the high proportion of patients with non-rapid *NAT2* genotypes described in this cohort, which has direct implications for isoniazid pharmacokinetics [19].

According to previous pharmacogenetic studies, the influence of *SLCO1B1* gene polymorphisms on rifampicin (RIF) pharmacokinetics and dosage appears to be limited and not consistently clinically significant. A large population pharmacokinetic analysis in Korean patients with tuberculosis found that, while the *SLCO1B1* rs4149056 genotype was statistically significantly associated with variations in rifampicin apparent clearance (CL/F), the overall impact on drug exposure was modest and insufficient to warrant clinical dosage adjustments beyond standard recommendations based on body weight; in that study, weight was the main covariate for dose optimization [20]. Similarly, another study that employed a population pharmacokinetic model in tuberculosis patients in Indonesia reported that, although the SLCO1B1*15 haplotype influenced rifampicin clearance, the magnitude of variability attributable to this gene was small compared to other factors (e.g., age and weight), and did not provide sufficient evidence to support genotype-guided dosing [21].

## 5. Conclusions

This study provides novel evidence on the distribution of polymorphisms in genes relevant to the pharmacokinetics of INH and RIF in Southern Peru. Our findings provide foundational data supporting the future integration of pharmacogenomics into TB management strategies to optimize therapeutic safety and efficacy. Future multicenter studies with larger sample sizes and clinical and pharmacokinetic correlations will help to consolidate these observations and advance a personalized medicine approach to tuberculosis.

## Figures and Tables

**Table 1:** Comparative pharmacogenetic profile of *NAT2* and *CYP2E1* in Peruvian tuberculosis patients.

Gene	Variant/Phenotype	Southern Peru (This Study) n (%)	Lima Cohort (Jaramillo-Valverde, 2023 [11]) n (%)
*NAT2*	Rapid acetylator	79 (20.%)	56/377 (14.9%)
	Intermediate acetylator	24 (68.6%)	142/377 (37.7%)
	Slow acetylator	4 11.4%)	179/377 (47.5%)
*CYP2E1*	c1/c1	≈22 (62.9%)	237/372 (63.7%)
	c1/c2	≈11 (31.4%)	115/372 (30.9%)
	c2/c2	≈2 (5.7%)	20/372 (5.4%)
*SLCO1B1*	rs4149056 variant	1–2 (2.9–5.7%)	Not evaluated

**Table 2:** Minor allele frequency (MAF) of relevant *NAT2* gene polymorphisms.

Gene	SNP (rsID)	Reference Allele	Alternative Allele	Genotyping	0/0 n (%)	0/1 n (%)	1/1 n (%)	Alternative Allele Frequency	MAF
*NAT2*	rs1041983	C	T	33	20 (60.6)	8 (24.2)	5 (15.2)	0.273	0.273
*NAT2*	rs1801280	T	C	34	20 (58.8)	8 (23.5)	6 (17.6)	0.294	0.294
*NAT2*	rs1799929	C	A/T	35	25 (71.4)	10 (28.6)	0 (0.0)	0.143	0.143
*NAT2*	rs1208	G	A	34	0 (0.0)	9 (26.5)	25 (73.5)	0.868	0.132
*NAT2*	rs1799931	G	A	33	22 (66.7)	8 (24.2)	3 (9.1)	0.212	0.212

Note: Genotypes are coded as follows: 0/0 (homozygous wild type), 0/1 (heterozygous), and 1/1 (homozygous mutant).

## Data Availability

The original data presented in the study are openly available in FigShare at https://doi.org/10.6084/m9.figshare.31115791 (raw data, accessed on 24 March 2026), https://doi.org/10.6084/m9.figshare.31112809 (processed and aligned sequences, accessed on 24 March 2026), and https://doi.org/10.6084/m9.figshare.31115791 (R-code used, accessed on 24 March 2026).

## Supplementary Materials

The following supporting information can be downloaded at: https://www.mdpi.com/article/10.3390/jpm16040184/s1, Figure S1. Uncropped figure for genotype and inferred haplotype profiles of *SLCO1B1*. Rows represent single-nucleotide polymorphisms (SNPs), ordered by hierarchical clustering based on genotype similarity, while columns correspond to individual samples. Genotypes are color-coded according to allele dosage: 0 = homozygous reference (yellow), 1 = heterozygous (green), and 2 = homozygous variant (blue); missing genotypes are shown in white.
