# Supplementary figures and images for: Population-Specific Pharmacogenomic Profiling of NAT2, CYP2E1, and SLCO1B1 in Tuberculosis Patients from Southern Peru: A Feasibility Pilot Study
Source: J Pers Med. 2026 Mar 29;16(4):184. doi: 10.3390/jpm16040184 (PMC13117989; doi:10.3390/jpm16040184)

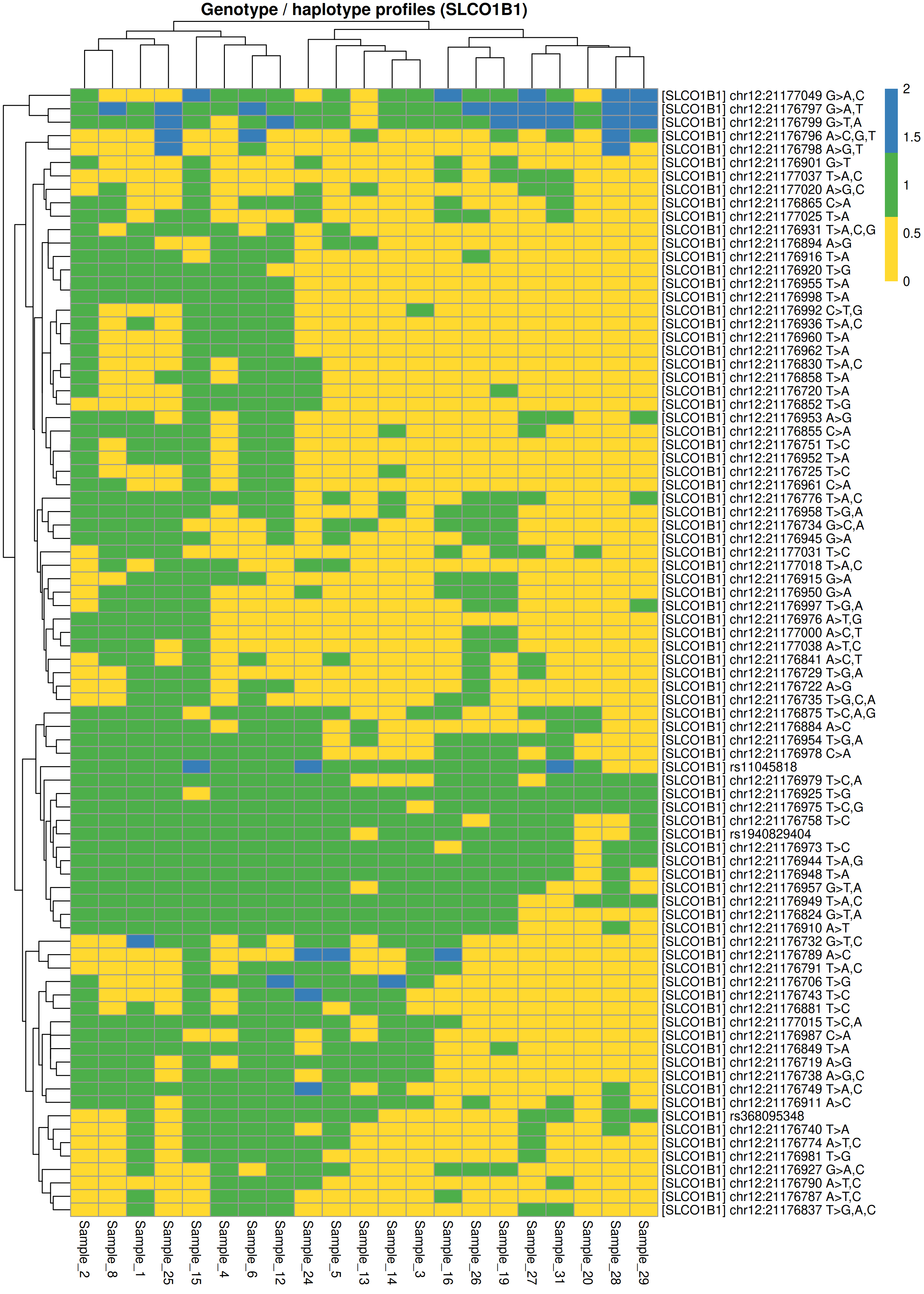

Supplement: Supplementary file 1 [file jpm-16-00184-s001.zip › jpm-4180182-supplementary.png]
